# Bortezomib plus rituximab versus rituximab in patients with high-risk, relapsed, rituximab-naïve or rituximab-sensitive follicular lymphoma: subgroup analysis of a randomized phase 3 trial

**DOI:** 10.1186/1756-8722-5-67

**Published:** 2012-10-22

**Authors:** Pier Luigi Zinzani, Nuriet K Khuageva, Huaqing Wang, Bernardo Garicochea, Jan Walewski, Achiel Van Hoof, Pierre Soubeyran, Dolores Caballero, Rena Buckstein, Dixie-Lee Esseltine, Panteli Theocharous, Christopher Enny, Eugene Zhu, Yusri A Elsayed, Bertrand Coiffier

**Affiliations:** 1 Institute of Hematology and Medical Oncology ‘L. & A. Seràgnoli’, University of Bologna, Bologna, Italy; 2 Hematology, SP Botkin Moscow City Hospital, Moscow, Russia; 3 Medical Oncology, Medical University Cancer Hospital, Tianjin, China; 4 Hematology, Hospital Sao Lucas, Porto Alegro, Brazil; 5 Hematology, Maria Sklodowska-Curie Memorial Institute and Oncology Centre, Warsaw, Poland; 6 Hematology, Hospital St Jan AV, Brugge, Belgium; 7 Medical Oncology, Institut Bergonié, Bordeaux, France; 8 Clinical Hematology, Hospital Clínico de Salamanca, Salamanca, Spain; 9 Medical Oncology & Hematology, Sunnybrook Regional Cancer Center, Toronto, Canada; 10 Clinical Development, Millennium Pharmaceuticals, Inc, Cambridge, MA, USA; 11 Oncology R&D, Janssen R&D, High Wycombe, UK; 12 Oncology R&D, Janssen R&D, Raritan, NJ, USA; 13 Hematology, Hospices Civils de Lyon, Lyon, France

**Keywords:** Bortezomib, Follicular, High risk, Lymphoma, Rituximab

## Abstract

**Background:**

The randomized phase 3 LYM3001 trial in relapsed follicular lymphoma (FL) demonstrated higher overall (ORR) and complete response (CR) rates and prolonged progression-free survival (PFS) with bortezomib-rituximab versus rituximab. We report findings in high-risk patients (FL International Prognostic Index [FLIPI] score ≥3, and high tumor burden by modified Groupe d’Etude des Lymphomas Folliculaires [GELF] criteria).

**Methods:**

Patients aged ≥18 years with grade 1/2 FL, ≥1 measurable lesion, and documented relapse or progression following prior therapy, rituximab-naïve or rituximab-sensitive, were enrolled at 164 centers in 29 countries across Europe, the Americas, and Asia-Pacific. Patients were randomized (1:1) to five 5-week cycles of bortezomib-rituximab (bortezomib 1.6 mg/m^2^, days 1, 8, 15, and 22, all cycles; rituximab 375 mg/m^2^, days 1, 8, 15, and 22, cycle 1, and day 1, cycles 2–5; N=336) or rituximab alone (N=340). Randomization was stratified by FLIPI score, prior rituximab, time since last dose of anti-lymphoma therapy, and geographical region. The primary endpoint of the study was PFS.

**Results:**

103 bortezomib-rituximab and 98 rituximab patients had high-risk FL. The ORR was 59% versus 37% (p=0.002), the CR/CRu rate was 13% versus 6% (p=0.145), and the durable response rate was 45% versus 26% (p=0.008) with bortezomib-rituximab versus rituximab. Median PFS was 9.5 versus 6.7 months (hazard ratio [HR] 0.667, p=0.012) with bortezomib-rituximab versus rituximab; median time to progression was 10.9 versus 6.8 months (HR 0.656, p=0.009); median time to next anti-lymphoma treatment was 14.8 versus 9.1 months (HR 0.762, p=0.103); and the 1-year Overall Survival rate was 83.1% versus 76.6%. Overall, 51% of bortezomib-rituximab and 32% of rituximab patients reported grade ≥3 adverse events, including neutropenia (18%, 6%), anemia (4%, 5%), diarrhea (8%, 0%), thrombocytopenia (5%, 2%), and sensory neuropathy (1%, 0%).

**Conclusions:**

High-risk FL patients treated with bortezomib-rituximab had significantly higher ORR and longer PFS than patients receiving rituximab alone, with greater clinical benefit than in the overall study population; additional toxicity was acceptable and did not affect treatment feasibility.

**Trial registration:**

The phase 3 LYM3001 trial is registered with ClinicalTrials.gov, with the identifier NCT00312845.

## Background

Follicular lymphoma (FL) is an incurable, indolent non-Hodgkin’s lymphoma (NHL) subtype that follows a relapsing course [1,2]. Although the introduction of new therapies and treatment approaches over the past couple of decades has improved progression-free survival (PFS) [3] and overall survival (OS) [4,5], prognosis differs significantly between patients according to multiple demographic and disease-related characteristics [5-10]. Adverse prognostic factors in FL include a high (≥3) FL International Prognostic Index (FLIPI) score, representing patients aged >60 years, and/or with stage III–IV disease, anemia, >4 involved nodal areas, and/or elevated lactate dehydrogenase (LDH) [7,10,11], which has been validated in the first-line setting [11] and shown to have prognostic value at first relapse [10]. In addition, another indicator of poor prognosis is a high tumor burden by modified Groupe d’Etude des Lymphomas Folliculaires (GELF) criteria [12], which includes involvement of ≥3 nodal sites of ≥3 cm diameter, any nodal/extranodal tumor mass of ≥7 cm diameter, splenomegaly, pleural effusion or peritoneal ascites, leukocytes <1.0 x 10^9^/L, or platelets <100 x 10^9^/L [12,13].

Patients with high-risk FL typically have a poor prognosis, including a higher probability of relapse and lower survival rates [7,10,11], and need better treatment options [10,14,15]. These patients may particularly benefit from novel, active regimens [10]. Rituximab is widely used in previously untreated and relapsed FL [16], having been shown to enhance the efficacy of chemotherapeutic regimens [17-19] and to improve PFS when used as maintenance therapy [3,20]. The proteasome inhibitor bortezomib has shown single-agent activity in heavily pretreated indolent lymphoma patients, with response rates of up to 50% [21-23], and bortezomib plus rituximab has been shown to be active and generally well tolerated in phase 2 studies in FL and other NHL subtypes [24-27].

The randomized, multicenter, international phase 3 LYM3001 trial compared bortezomib plus rituximab with rituximab alone in 676 patients with relapsed, rituximab-naïve or rituximab-sensitive FL [28]. Results from the overall study population showed that the addition of bortezomib resulted in prolonged PFS (primary endpoint; median 12.8 vs. 11.0 months, hazard ratio [HR] 0.822; *P*=0.039), a higher overall response rate (ORR; 63% vs. 49%, *P*<0.001), a higher complete response (CR)/unconfirmed CR (CRu) rate (25% vs. 18%, *P*=0.035), and more durable responses. In this ad-hoc subgroup analysis of LYM3001, we report the activity and safety of rituximab ± bortezomib in patients with high-risk disease, defining ‘high-risk’ patients as those having both a high (≥3) FLIPI score and high tumor burden by modified GELF criteria [12], both of which were independent pre-defined subgroups.

## Results

### Patients

In the overall study population, 139 patients in the bortezomib-rituximab arm and 140 patients in the rituximab arm had high FLIPI score (≥3), and 185 and 179 patients, respectively, had high tumor burden by modified GELF criteria [12]. Of these patients, 103 in the bortezomib-rituximab arm and 98 in the rituximab arm had high-risk FL per the definition for this subgroup analysis (both FLIPI score ≥3 and high tumor burden by modified GELF criteria [12]). High-risk patient demographics and baseline disease characteristics in the intention-to-treat (ITT) population are shown in Table 1. Demographics were consistent with those in the overall study population; disease characteristics reflected the high-risk nature of the patients compared with the overall study population [28]. Disease characteristics and prior therapy exposure were generally balanced between arms except for Ann Arbor staging and sex, with higher proportions of patients with stage IV disease and males in the bortezomib-rituximab arm. 

**Table 1:** Baseline characteristics of LYM3001 patients with high-risk FL (ITT population)

	**Bortezomib-rituximab (N=103)**	**Rituximab (N=98)**
Median age, years (range)	61 (38–83)	60 (21–84)
Aged >65 years, n (%)	31 (30)	25 (26)
Male, n (%)	53 (51)	42 (43)
Race, n (%)		
White	78 (76)	65 (66)
Asian	20 (19)	29 (30)
Other	5 (5)	4 (4)
Region, n (%)		
USA and Canada	8 (8)	10 (10)
European Union	34 (33)	28 (29)
Rest of the World*	61 (59)	60 (61)
ECOG performance status, n (%)		
0	36 (35)	35 (36)
1	56 (54)	52 (53)
2	11 (11)	11 (11)
Ann Arbor Stage, n (%)		
II	0	1 (1)
III	36 (35)	45 (46)
IV	67 (65)	52 (53)
High tumor burden by modified GELF criteria [12], n (%)	103 (100)	98 (100)
High (≥3) FLIPI score [7], n (%)	103 (100)	98 (100)
Elevated serum LDH, n (%)	70 (68)	61 (62)
Median time from initial diagnosis, months (range)	35.8 (2–233)	38.5 (1–197)
Number of prior lines of therapy, n (%)		
1	43 (42)	35 (36)
2	25 (24)	31 (32)
≥3	35 (34)	32 (32)
Common prior therapies, n (%)		
CHOP	41 (40)	40 (41)
CVP	35 (34)	23 (23)
Single-agent rituximab	17 (17)	13 (13)
Chlorambucil-prednisone	9 (9)	14 (14)
R-CHOP	10 (10)	12 (12)
R-CVP	9 (9)	12 (12)
Single-agent chlorambucil	10 (10)	10 (10)
Any prior rituximab therapy, n (%)	40 (39)	40 (41)
>1 year since last FL treatment, n (%)	53 (51)	48 (49)
Creatinine clearance >30–60 mL/min, n (%)	18 (17)	24 (24)

*Including 21%/12% from Russia, 10%/14% from People’s Republic of China, 9%/14% from India, and 9%/11% from Brazil in the bortezomib-rituximab/rituximab arms.

*ECOG*, Eastern Cooperative Oncology Group; *GELF*, Groupe d’Etude des Lymphomas Folliculaires; *FLIPI*, Follicular Lymphoma International Prognostic Index; *LDH,* lactate dehydrogenase.

Patient disposition and treatment exposure in the safety population are shown in Table 2. One patient with high-risk FL in the bortezomib-rituximab arm was not treated and was excluded from the safety population. Treatment exposure was generally comparable with that in the overall study population [28], with patients in both arms receiving a median of five cycles. However, slightly lower proportions of patients in each arm completed all five cycles in this high-risk population (64% and 53% in the bortezomib-rituximab and rituximab arms, respectively) compared with in the overall population (72% and 71%, respectively) [28], reflecting that these patients were more challenging to treat. Among the reasons for discontinuing treatment prior to completing five cycles, disease progression was less common (25% vs. 39%) but adverse events (AEs) more common (8% vs. 2%) in patients receiving bortezomib-rituximab versus rituximab. 

**Table 2:** Patient disposition and treatment exposure (safety population)

	**Bortezomib-rituximab (N=102)**	**Rituximab (N=98)**
Median number of cycles, n (range)	5 (1–5)	5 (1–5)
Median treatment duration, weeks (range)	25 (5–33)	25 (5–27)
Patients completing all 5 cycles, n (%)	65 (64)	52 (53)
Patients discontinuing prior to completing 5 cycles, n (%), due to:	37 (36)	46 (47)
Disease progression	26 (25)	38 (39)
AEs	8 (8)	2 (2)
Treatment-related	6 (6)	0
Death	1 (1)	1 (1)
Patient choice	2 (2)	4 (4)
Other reasons	0	1 (1)
Mean rituximab relative dose intensity, %	98	96
Mean bortezomib relative dose intensity, %	90	NA

*AEs*, adverse events; *NA*, not applicable.

### Efficacy

Bortezomib-rituximab resulted in a higher ORR and CR/CRu rate than rituximab in patients with high-risk FL (Table 3). Similarly, the rate of durable (≥6 months) response was higher, and median duration of response (DOR) in patients with CR/CRu was prolonged with bortezomib-rituximab, although median DOR in all responding patients appeared somewhat shorter.

**Table 3:** Best response to treatment and durability of response (response-evaluable population)

	**Bortezomib-rituximab (N=96)**	**Rituximab (N=95)**	**Odds ratio (95% CI)**	**P-value**
ORR, n (%)	57 (59)	35 (37)	0.399 (0.223, 0.715)	0.002
CR/CRu*, n (%)	12 (13)	6 (6)	0.472 (0.169, 1.314)	0.145
PR, n (%)	45 (47)	29 (31)	–	–
Stable disease, n (%)	24 (25)	32 (34)	–	–
Progressive disease, n (%)	15 (16)	28 (29)	–	–
Durable (≥6 months) response, n (%)	43 (45)	25 (26)	0.440 (0.240, 0.809)	0.008
Median DOR, months	10.4	12.1	–	–
Median DOR (CR/CRu*), months	16.5	10.5	–	–

*Radiologic CR/CRu verified by bone marrow and lactate dehydrogenase.

*CI*, confidence interval; *CR*, complete response; *CRu*, unconfirmed CR; *DOR*, duration of response; *ORR*, overall response rate; *PR*, partial response.

In patients with high FLIPI score (≥3) regardless of tumor burden, ORR was 64% versus 45% (odds ratio 0.459 [95% confidence interval (CI) 0.279, 0.756], p=0.002), CR/CRu rate was 20% versus 10% (odds ratio 0.446 [95% CI 0.217, 0.916], p=0.026), and durable response rate was 50% versus 32% (odds ratio 0.459 [95% CI 0.277, 0.761], p=0.002) with bortezomib-rituximab versus rituximab; in patients with high tumor burden regardless of FLIPI score, ORR was 60% versus 41% (odds ratio 0.468 [95% CI 0.306, 0.717], p<0.001), CR/CRu rate was 19% versus 10% (odds ratio 0.476 [95% CI 0.254, 0.891], p=0.019), and durable response rate was 45% versus 32% (odds ratio 0.581 [95% CI 0.377, 0.897], p=0.014) with bortezomib-rituximab versus rituximab.

**Figure 1:**
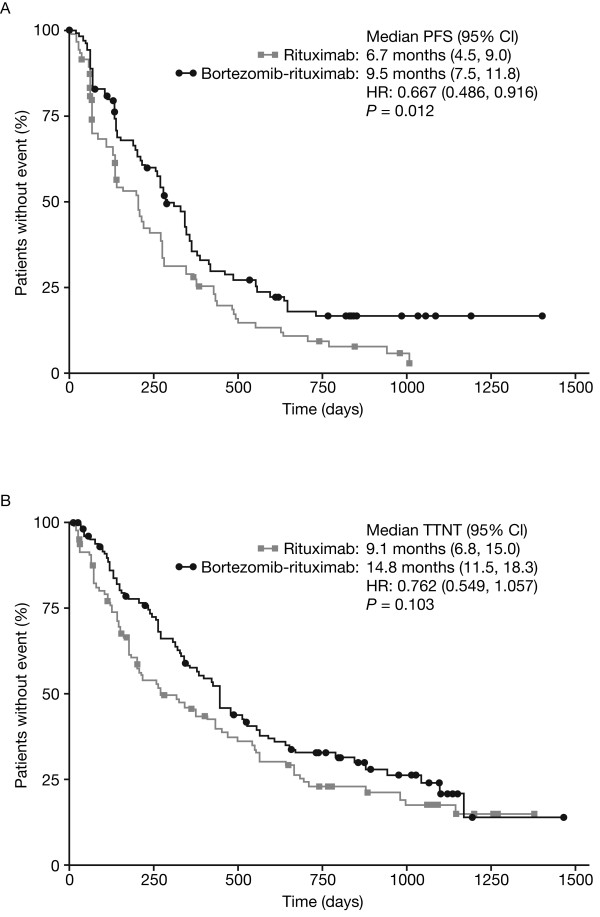
(A) PFS and (B) TTNT by treatment arm in patients with high-risk FL.

With a median follow-up of 35.2 months in each arm in the ITT population of high-risk patients, median PFS was 9.5 months in the bortezomib-rituximab arm versus 6.7 months in the rituximab arm (HR 0.667, p=0.012) (Figure 1A). Other outcomes were also longer with bortezomib-rituximab versus rituximab, including: time to progression (TTP; median 10.9 [95% CI 8.6, 11.8] vs. 6.8 [95% CI 4.5, 9.1] months; HR 0.656, p=0.009); time to next anti-lymphoma treatment (TTNT; median 14.8 vs. 9.1 months; HR 0.762, p=0.103) (Figure 1B); and treatment-free interval (TFI; median 9.2 [95% CI 6.2, 13.0] vs. 5.6 [95% CI 2.6, 10.4] months).

In patients with high FLIPI score (≥3) regardless of tumor burden, median PFS was 11.4 versus 7.9 months (HR 0.707, p=0.013), median TTP was 11.5 versus 9.0 months (HR 0.688, p=0.009), median TTNT was 17.1 versus 14.4 months (HR 0.760, p=0.067), and median TFI was 12.8 versus 9.8 months (HR 0.788, p=0.110) with bortezomib-rituximab versus rituximab; in patients with high tumor burden regardless of FLIPI score, median PFS was 11.3 versus 8.4 months (HR 0.751, p=0.019), median TTP was 11.4 versus 8.9 months (HR 0.747, p=0.018), median TTNT was 16.9 versus 13.5 months (HR 0.751, p=0.024), and median TFI was 11.1 versus 8.5 months (HR 0.780, p=0.050) with bortezomib-rituximab versus rituximab.

The 1-year OS rate was 83.1% (95% CI 75.8, 90.4) versus 76.6% (95% CI 68.0, 85.2) (HR 0.907, p=0.657) with bortezomib-rituximab and rituximab, respectively. Median OS was 37.8 (95% CI 31.9, not estimable) versus 41.5 (95% CI 25.0, not estimable) months, with 43 (42%) and 41 (42%) patients having died in the bortezomib-rituximab and rituximab arms, respectively. In patients with high FLIPI score (≥3) regardless of tumor burden, the 1-year OS rate was 85.1% versus 82.8% (HR 0.952, p=0.802) with bortezomib-rituximab versus rituximab; respective rates in patients with high tumor burden regardless of FLIPI score were 87.2% versus 84.3% (HR 0.981, p=0.919).

### Safety

The safety profiles of bortezomib-rituximab and rituximab in patients with high-risk FL are summarized in Table 4. The rates of grade ≥3 AEs (51% vs. 32%), serious AEs (22% vs. 16%), and AEs leading to treatment discontinuation (8% vs. 2%) were higher with bortezomib-rituximab versus rituximab.

**Table 4:** Safety profiles in high-risk FL patients (safety population)

**AE, n (%)**	**Bortezomib-rituximab (N=102)**		**Rituximab (N=98)**	
Any AE	97 (95)		88 (90)	
Any related AE	91 (89)		64 (65)	
Any rituximab-related AE	70 (69)		64 (65)	
Any bortezomib-related AE	85 (83)		NA	
Any grade ≥3 AE	52 (51)		31 (32)	
Any serious AE	22 (22)		16 (16)	
Any related serious AE	14 (14)		4 (4)	
AE leading to treatment withdrawal	8 (8)		2 (2)	
Deaths due to AEs	2 (2)		1 (1)	
**Most common AEs**	**All grades**	**Grade ≥3**	**All grades**	**Grade ≥3**
Diarrhea	50 (49)	8 (8)	11 (11)	0
Pyrexia	30 (29)	0	16 (16)	1 (1)
Fatigue	24 (24)	1 (1)	10 (10)	0
Neutropenia	21 (21)	18 (18)	11 (11)	6 (6)
Febrile neutropenia	1 (1)	1 (1)	3 (3)	3 (3)
Nausea	19 (19)	1 (1)	11 (11)	0
Abdominal pain	18 (18)	2 (2)	9 (9)	1 (1)
Decreased appetite	18 (18)	0	4 (4)	0
Infections	58 (57)	16 (16)	27 (28)	7 (7)
Upper respiratory tract	16 (16)	3 (3)	6 (6)	1 (1)
Herpes zoster	16 (16)	4 (4)	3 (3)	1 (1)
Pneumonia	6 (6)	3 (3)	4 (4)	3 (3)
Peripheral edema	15 (15)	2 (2)	13 (13)	2 (2)
Asthenia	15 (15)	1 (1)	11 (11)	4 (4)
Vomiting	15 (15)	0	11 (11)	2 (2)
Constipation	15 (15)	0	8 (8)	0
Peripheral sensory neuropathy	15 (15)	1 (1)	0	0
Dyspnea	13 (13)	1 (1)	10 (10)	4 (4)
Anemia	12 (12)	4 (4)	12 (12)	5 (5)
Thrombocytopenia	12 (12)	5 (5)	8 (8)	2 (2)
Leukopenia	7 (7)	3 (3)	12 (12)	2 (2)

*AE,* adverse event; *NA*, not applicable.

Table includes the most commonly reported AEs (≥15% at any grade or ≥3% grade ≥ 3 in either arm).

The most common AEs occurring at any grade in ≥15% of patients in either arm, or at grade ≥3 in ≥3% of patients in either arm, are also listed in Table 4. AEs were mostly mild or moderate (grade 1 or 2), with only neutropenia (18% and 6% in the bortezomib-rituximab and rituximab arms, respectively), infections (16%, 7%), anemia (4%, 5%), diarrhea (8%, 0%), and thrombocytopenia (5%, 2%) being reported at grade ≥3 in ≥5% patients in either arm. As shown in Table 4, peripheral sensory neuropathy was reported in 15 patients in the bortezomib-rituximab arm, and was grade 1 in 10 patients, grade 2 in four patients, and grade 3 in one patient. Additionally, one patient in this arm experienced grade 2 peripheral motor neuropathy. The only serious AEs reported in ≥2% of patients overall were pneumonia (5% in bortezomib-rituximab arm, 1% in rituximab arm), febrile neutropenia (1%, 3%), neutropenia (2%, 2%), pyrexia (3%, 1%), and diarrhea (2%, 1%).

Two deaths due to treatment-related AEs were reported in the bortezomib-rituximab arm. These were due to: septic shock (considered possibly related to both bortezomib and rituximab); and bilateral pneumonia (considered possibly related to bortezomib but not rituximab) as well as acute respiratory failure and acute cardiac failure, both considered unrelated to treatment [28]. In the rituximab arm, the one death due to an AE was due to meningitis (considered unrelated to treatment) [28].

## Discussion

This ad-hoc subgroup analysis of the phase 3 LYM3001 study [28] of relapsed FL patients with the high-risk features of both high FLIPI score (≥3) and high tumor burden by modified GELF criteria [12] demonstrated a number of key efficacy findings. In particular, we showed that patients with high-risk FL treated with bortezomib-rituximab had significantly higher response rates and longer PFS than patients receiving rituximab alone. Furthermore, while overall median DOR appeared similar between arms, the durability of CR/CRu appeared enhanced with the addition of bortezomib in this high-risk patient population. The higher rate and durability of CR/CRu with bortezomib-rituximab is notable, as CR has been associated with improved outcomes in FL [29]. Additionally, the bortezomib-rituximab combination was associated with a significantly longer TTP, and a longer TTNT and associated TFI compared with rituximab alone. No significant difference was seen in OS between arms, as might be expected after a median follow-up of 3 years in a patient population with a typically indolent disease course and a reported median OS from diagnosis of up to approximately 14 years [4]. However, the 1-year OS rate appeared slightly higher in the bortezomib-rituximab arm, possibly due to an increased rate of progressive disease-related deaths in the rituximab arm during this period.

Notably, the consistent relative clinical benefit with bortezomib-rituximab versus rituximab in this subgroup of high-risk patients was greater than that seen in the overall study population [28], as indicated by HRs more in favor of bortezomib-rituximab. For example, the HR for PFS benefit with bortezomib-rituximab versus rituximab was 0.667 in these high-risk patients, compared with 0.882 in the overall study population [28]. Similarly, the odds ratios for ORR (0.399 vs. 0.569), CR/CRu rate (0.472 vs. 0.665), and durable (≥6 months) response rate (0.440 vs. 0.608), and the HRs for TTP (0.656 vs. 0.808), TTNT (0.762 vs. 0.799), and OS (0.907 vs. 0.971) indicated greater clinical benefit with bortezomib-rituximab versus rituximab in the high-risk subgroup versus the overall population. All these data suggest that the addition of bortezomib might thus provide greater tumor reduction and subsequent sustained clinical benefit in these patients who have a greater disease burden or more proliferative FL.

The patient population included in these analyses represented a true ‘high-risk’ subset of FL patients because they presented with both a high (≥3) FLIPI score and high tumor burden, both of which are factors that define cohorts of FL patients with disease that is high-risk in nature. The poor prognostic nature of each of these factors is supported by our efficacy analyses in the specific individual subsets with high FLIPI score or with high tumor burden, which demonstrated poorer outcomes compared with in the overall study population [28]; furthermore, our efficacy findings suggest that the presence of both high FLIPI score and high tumor burden defines a population with poorer prognosis than either factor alone. In supportive evidence from the literature, a FLIPI score of ≥3 has been associated with poor prognosis in terms of PFS and OS in a number of studies and analyses in both previously untreated and relapsed FL patients treated with various different regimens and modalities [7,9-11,30,31]. Similarly, the presence of a high tumor burden has been shown to be a significant poor prognostic factor [13]. As previously noted [28], there is no standard of care for relapsed FL, with various different approaches recommended as possible therapeutic options in guidelines from the European Society for Medical Oncology [32] and the US National Comprehensive Cancer Network [33], including rituximab-chemotherapy and rituximab maintenance [3,16]. However, there is no specific management strategy defined for FL patients with high FLIPI score and/or high tumor burden, and thus identifying treatment options offering relatively greater clinical benefit in patients with these poor prognostic factors is of interest.

It is important to note that, per protocol, none of the patients in LYM3001 were rituximab-refractory, another factor representing a high-risk, difficult-to-treat population. Nevertheless, non-clinical studies provide a rationale for the potential utility of the bortezomib-rituximab combination in this patient subset; some preclinical data suggest that addition of bortezomib might be useful for overcoming resistance in FL and for resensitizing patients to treatment, including rituximab [34-36].

Regarding the safety profiles of bortezomib-rituximab and rituximab alone in this subgroup analysis of patients with high-risk FL, our findings reflect those in the overall study population but with somewhat elevated rates of toxicity in these high-risk patients [28]. As would be expected, bortezomib-rituximab was associated with additional toxicity and rates of AEs were higher in the combination arm compared with in the rituximab arm; however this did not affect treatment feasibility, with patients receiving a median of five cycles of treatment in both arms and a higher proportion completing all five planned cycles in the bortezomib-rituximab arm than in the rituximab arm. As reported previously [28], the addition of bortezomib to rituximab was associated with peripheral neuropathy AEs, with 15% of patients in the bortezomib-ritxumab arm experiencing peripheral sensory neuropathy and 1% experiencing peripheral motor neuropathy; however, only one grade 3 AE, of peripheral sensory neuropathy, was reported in these high-risk patients. As noted previously [28], this rate and severity of peripheral neuropathy appeared limited compared with previous reports in myeloma, which was likely associated with the use of a weekly, instead of a twice-weekly, bortezomib dosing regimen.

## Conclusions

In conclusion, bortezomib-rituximab appears an effective regimen for the management of patients with relapsed FL, offering clinical benefit compared with rituximab alone in this high-risk subgroup of patients with the poor prognostic features of high FLIPI score (≥3) and high tumor burden. Further prospective validation of these findings is warranted; an Eastern Cooperative Oncology Group (ECOG) phase 2 study is currently investigating bendamustine-rituximab with or without bortezomib in patients with either high FLIPI score (≥3) or high tumor burden by GELF criteria (NCT01216683). Ongoing biomarker analyses of the LYM3001 study may enable identification of specific patient subgroups deriving greater benefit from bortezomib-rituximab versus rituximab treatment.

## Methods

### Patients and study design

The LYM3001 study design has been reported previously [28]. LYM3001 was conducted at 164 centers in 29 countries across Europe, the Americas, and Asia-Pacific [28]; patients were enrolled between April 10, 2006, and August 12, 2008, and clinical data cut-off was June 15, 2010. Patients aged ≥18 years were enrolled in the study if they met all of the following key inclusion criteria: grade 1/2 FL (WHO criteria [37]) with ≥1 measurable lesion; documented relapse or progression following prior therapy; rituximab-naïve or rituximab-sensitive (defined as a response to and TTP of ≥6 months with prior rituximab-containing therapy); ECOG performance status ≤2; and no active central nervous system lymphoma. Patients were excluded if they had grade ≥2 peripheral neuropathy or neuropathic pain, or clinical evidence of a transformation to an aggressive lymphoma, or had received prior bortezomib. Review boards at all participating institutions approved the study, which was conducted according to the Declaration of Helsinki and International Conference on Harmonization Guidelines for Good Clinical Practice. All patients provided written informed consent.

Patients were randomized (1:1) to receive five 5-week cycles consisting of bortezomib 1.6 mg/m^2^ on days 1, 8, 15, and 22, plus rituximab 375 mg/m^2^ on days 1, 8, 15, and 22 in cycle 1 and on day 1 in cycles 2–5; or rituximab alone on the same schedule. Randomization was stratified by FLIPI score (0–1 [low], 2 [intermediate], ≥3 [high]), prior rituximab therapy (yes, no), time since last dose of anti-lymphoma therapy (≤1, >1 year), and geographical region (United States/Canada, European Union, rest of the world). Patients were assigned based on a computer-generated randomization schedule that was pre-prepared by the study sponsor, as previously described [28].

The primary endpoint of the study was PFS; the secondary endpoints included ORR, CR rate, DOR, TTP, 1-year OS rate, and safety/tolerability. TTNT and TFI were additional predefined endpoints of clinical benefit. As reported previously [28], preplanned efficacy analyses were conducted in patient subgroups defined according to age, sex, ethnic origin, FLIPI score, prior rituximab, prior lines of therapy, time since last dose of antilymphoma treatment, disease stage, and tumor burden.

### Assessments

Patients’ FLIPI score and tumor burden were recorded at screening based on the respective constitutive clinical parameters. An independent radiology committee (IRC) assessed tumor response and progression using modified International Working Group Response Criteria [38]. Computed tomography (CT) scans with contrast (plus magnetic resonance imaging [MRI] if required) were performed at baseline, and every 10 weeks during and following treatment until disease progression or death; bone marrow aspiration and biopsy were performed for confirmation of CR. Upon disease progression, patients were followed every 3 months for survival, and subsequent anti-lymphoma therapy was recorded. AEs were graded according to the National Cancer Institute’s Common Terminology Criteria for AEs (NCI CTCAE) version 3.0.

### Statistical analysis

For the overall LYM3001 study, the planned sample size was 670 patients (accrual of 676 patients achieved), and the target number of PFS events was 514 [28]. The study was analyzed early, after 440 PFS events, per Independent Data Monitoring Committee recommendations as the planned 514 events would probably have been unachievable within a reasonable timeframe due to very low ongoing accumulation of PFS events [28].

PFS, TTP, TTNT, and OS were analyzed in the ITT population, with distributions estimated using the Kaplan-Meier method. Unstratified log-rank tests were used for comparisons between the bortezomib-rituximab and rituximab arms for high-risk patients and for patients with high FLIPI score or high tumor burden (*P*-values <0.05 were considered statistically significant). A Cox regression model was used for estimation of HRs and 95% CIs. ORR, CR rate, and DOR were analyzed in the response-evaluable population, which included all patients who received at least one dose of study drug, had at least one measurable tumor mass at baseline, and had at least one post-baseline response assessment by IRC. DOR was analyzed by the Kaplan-Meier method. The safety population comprised all patients who received at least one dose of study drug. Statistical analyses were performed with SAS software, version 9.1.3. The LYM3001 study is registered with ClinicalTrials.gov, number NCT00312845.

## Abbreviations

AE: Adverse event; CI: Confidence interval; CR: Complete response; CRu: Unconfirmed complete response; CT: Computed tomography; DOR: Duration of response; FL: Follicular lymphoma; FLIPI: Follicular Lymphoma International Prognostic Index; GELF: Groupe d’Etude des Lymphomas Folliculaires; HR: Hazard ratio; IRC: Independent radiology committee; ITT: Intention-to-treat; LDH: Lactate dehydrogenase; MRI: Magnetic resonance imaging; NCI-CTCAE: National Cancer Institute Common Terminology Criteria for Adverse Events; NHL: Non-Hodgkin’s lymphoma; ORR: Overall response rate; OS: Overall survival; PFS: Progression-free survival; TFI: Treatment-free interval; TTNT: Time to next anti-lymphoma treatment; TTP: Time to progression; WHO: World Health Organization; PR: partial response.

## Competing interests

PLZ: no competing interests to declare; NKK: no competing interests to declare; HW: no competing interests to declare; BG: no competing interests to declare; JW: research and travel grants, and lecture honoraria, Roche; AVH: no competing interests to declare; PS: no competing interests to declare; DC: no competing interests to declare; RB: consultancy, Celgene, Novartis, GSK; DLE: employment, Millennium Pharmaceuticals, Inc., stock ownership, Johnson & Johnson; PT: employment, Janssen, stock ownership, Johnson & Johnson; CE: employment, Janssen, stock ownership, Johnson & Johnson; EZ: employment, Janssen, stock ownership, Johnson & Johnson; YAE: employment, Janssen, stock ownership, Johnson & Johnson; BC: consulting fees/honoraria, travel support, Johnson & Johnson, consultancy, Roche, Amgen, Sanofi, Pfizer, Millennium Pharmaceuticals, Inc., Celgene, Pharmacyclics, MedImmune, and CTI.

## Authors’ contributions

D-LE, PT, YAE, and BC were involved in the design of the study. PLZ, NKK, HW, BG, JW, AVH, PS, DC, RB, and BC recruited and treated patients for this study. PLZ, NKK, HW, BG, JW, AVH, PS, DC, RB, PT, CE, EZ, YAE, and BC were involved in data collection and collation. EZ did statistical analyses of the data. PLZ, D-LE, PT, CE, YAE, and BC were involved in interpreting the data. PLZ, PT, CE, EZ, YAE, and BC were involved in writing the draft of the report. All authors reviewed the draft manuscript and provided critical feedback. All authors reviewed and approved the final draft of the manuscript.

## Financial support

This research was funded by Johnson & Johnson Pharmaceutical Research & Development, L.L.C. and Millennium Pharmaceuticals, Inc.
